# Surgical Advances in Osteosarcoma

**DOI:** 10.3390/cancers13030388

**Published:** 2021-01-21

**Authors:** Marcus J. Brookes, Corey D. Chan, Bence Baljer, Sachin Wimalagunaratna, Timothy P. Crowley, Maniram Ragbir, Alistair Irwin, Zakareya Gamie, Thomas Beckingsale, Kanishka M. Ghosh, Kenneth S. Rankin

**Affiliations:** 1 Translational and Clinical Research Institute, Newcastle University, Newcastle NE1 7RU, UK; corey.chan@newcastle.ac.uk (C.D.C.); bence.baljer2@nhs.net (B.B.); sachin.wimalagunaratna1@nhs.net (S.W.); zakareya.gamie@newcastle.ac.uk (Z.G.); 2 North of England Bone and Soft Tissue Tumour Service, Newcastle upon Tyne NHS Hospitals Trust, Newcastle upon Tyne NE7 7DN, UK; timothy.crowley1@nhs.net (T.P.C.); maniram.ragbir@nhs.net (M.R.); alistair.irwin3@nhs.net (A.I.); t.beckingsale@nhs.net (T.B.); kanishka.ghosh1@nhs.net (K.M.G.); 3 Department of Plastic Surgery, Royal Victoria Infirmary, Newcastle upon Tyne NE1 4LP, UK

**Keywords:** osteosarcoma, sarcoma, surgery

## Abstract

**Simple Summary:**

Osteosarcoma (OS) is the most common bone cancer in children. OS most commonly arises in the legs, but can arise in any bone, including the spine, head or neck. Along with chemotherapy, surgery is a mainstay of OS treatment and in the 1990s, surgeons began to shift from amputation to limb-preserving surgery. Since then, improvements in imaging, surgical techniques and implant design have led to improvements in functional outcomes without compromising on the cancer outcomes for these patients. This paper summarises these advances, along with a brief discussion of future technologies currently in development.

**Abstract:**

Osteosarcoma (OS) is the most common primary bone cancer in children and, unfortunately, is associated with poor survival rates. OS most commonly arises around the knee joint, and was traditionally treated with amputation until surgeons began to favour limb-preserving surgery in the 1990s. Whilst improving functional outcomes, this was not without problems, such as implant failure and limb length discrepancies. OS can also arise in areas such as the pelvis, spine, head, and neck, which creates additional technical difficulty given the anatomical complexity of the areas. We reviewed the literature and summarised the recent advances in OS surgery. Improvements have been made in many areas; developments in pre-operative imaging technology have allowed improved planning, whilst the ongoing development of intraoperative imaging techniques, such as fluorescent dyes, offer the possibility of improved surgical margins. Technological developments, such as computer navigation, patient specific instruments, and improved implant design similarly provide the opportunity to improve patient outcomes. Going forward, there are a number of promising avenues currently being pursued, such as targeted fluorescent dyes, robotics, and augmented reality, which bring the prospect of improving these outcomes further.

## 1. Introduction

Osteosarcoma (OS) is the most common paediatric bone cancer [1,2]. It occurs most frequently in adolescents, with a second peak in those aged 60–80 years old, and is more common in males [2,3]. Those under 40 years of ages generally do better [2]; Whelan et al. found a 5 year survival of 56%, rising to 62% in those under 10 years old [3]. UK [4], European (ESMO-PaedCan-EUROCAN) [5] and North American (NCCN) [6] guidelines for localised OS (and metastatic disease in which all sites are resectable) advise neoadjuvant chemotherapy, followed by surgery and then adjuvant chemotherapy. Typical induction and adjuvant chemotherapy is a combination of high-dose methotrexate, doxorubicin and cisplatin; the degree of histological response to chemotherapy is associated with overall survival [3]. While the surgical management varies depending on the characteristics of the tumour, the aim is to achieve surgical resection with wide margins [4].

OS occurs most commonly at the end of long bones in the metaphyseal region, often extending into the epiphysis [2,3,7]. The pathogenesis of OS is thought to be due to an oncogenic event in osteoblast precursor cells which are dividing rapidly during skeletal growth. This explains why the highest incidence of OS is at anatomical sites in long bones that contribute the most to limb length gain during the growth spurt i.e., the distal femur and proximal tibia in the leg and the proximal humerus in the arm [2,3,8]. The most common histological subtypes of primary OS are shown in Table 1 [9,10]; the majority of the remaining cases are secondary OS, usually arising secondary to Paget’s disease or radiotherapy.

Many patients present with tumours that have destroyed the cortex and extruded out into the adjacent soft tissues, often in proximity to critical structures, such as important nerves or blood vessels. Resecting the tumour with clear margins, whilst preserving these structures, and the adjacent joint, is therefore challenging. This is important as histologically positive or close margins are associated with increased local recurrence [11] and decreased survival [12,13]. Historically, amputation rates were high, however there has been a shift towards limb salvage surgery, occurring in the 1990s [14]. This shift has not been associated with a decrease in survival, something that has likely been possible due to the introduction of improved chemotherapy regimens around this time [15]. The transition to limb salvage surgery has been beneficial to many patients, with evidence of improved functional outcomes compared to amputation, particularly with regards to physical function [16].

Given the importance of achieving clear margins for prognostic benefit, yet the potential detriment to the patient’s functional abilities, if too much normal tissue is resected, it is pivotal to develop surgical practice in order to maximise oncological outcome without sacrificing functional outcomes. The main avenues to explore are imaging and surgical techniques. Improved imaging pre-operatively allows improved planning of the surgery to be performed, while intraoperative imaging may guide surgeons to more accurately identify pertinent anatomical structures and avoid straying into the tumour during the procedure. This not only increases the likelihood of clear margins, but should increase confidence in leaving behind as much normal tissue as possible, in turn benefiting functional outcomes. Improved surgical techniques, such as computer assisted navigation surgery (CANS), along with patient specific jigs, allow these plans to be more accurately enacted. Benefits to functional outcomes can then further be increased by improved implants. The current progress in the above domains will be discussed in the following sections.

## 2. Pre-Operative Imaging

### 2.1. Current Guidelines

UK [4], European (ESMO-PaedCan-EUROCAN) [5] and North American (NCCN) [6] guidelines stipulate that, prior to any therapeutic input, OS first require plain radiographs, usually in two planes. It is recommended that this is then followed by magnetic resonance imaging (MRI) of the whole anatomical compartment, as well as the adjacent joints to assess for skip lesions. Computerised tomography (CT) scans are routinely only utilised for staging of metastases, or, infrequently, at the primary site when there is diagnostic uncertainty, if the MRI is contraindicated or as an adjunct in pelvic tumours. Certain centres also stage using a Positron emission tomography (PET) combined with a CT scan for the assessment of occult bone and soft tissue metastases; PET/CT and/or bone scans are specifically recommended within the NCCN guidelines [6]. Since chemotherapy may reduce the size of the primary tumour, surgical margins are usually planned from pre-treatment scans in order to mitigate the risk of leaving any viable tumour behind. 

### 2.2. Magnetic Resonance Imaging and Positron Emission Tomography (MRI/PET) Approach

**Figure 1:**
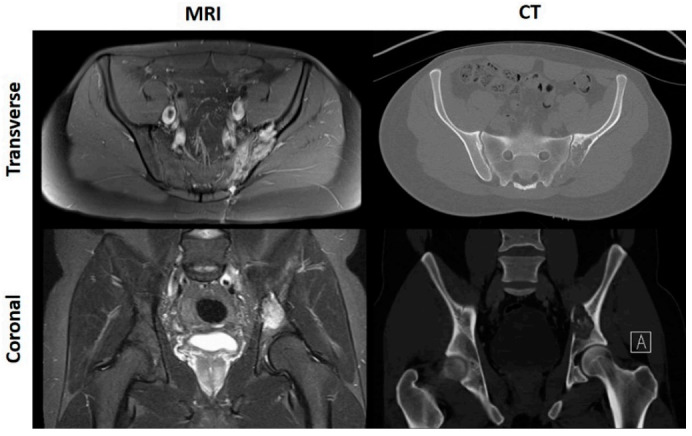
MRI and CT scans of a patient with left sided pelvic OS.

CT scans are excellent at delineating bony anatomy, thereby picking up pathological fractures and assessing ossification and calcification more accurately [17,18]. Nevertheless, as the aforementioned guidelines allude to, MRI is the most accurate tool for the determination of tumour limits within and outside of the bone, as it best defines medullary extent and soft tissue components [19,20,21,22,23]; demonstrated by Figure 1.

PET scans, which are able to detect metabolic activity, are beginning to show immense value within the field of oncology [24,25,26,27,28,29,30]. By combining images obtained from the PET scans with CT and MRI images, the metabolic and biochemical activity of the tumour can be overlaid on the anatomical structure to more precisely determine tumour margins [10]. MRI/PET also has the benefit of reducing radiation exposure when compared to CT [10].

In addition to more accurate surgical margins, MRI/PET can also detect systemic tumour involvement, local recurrence and metastasis after treatment [10]. Furthermore, combined information from PET and MRI scans has shown to be predictive of histological response to neoadjuvant chemotherapy in OS [31], even after a single course of chemotherapy [32].

There are however two main drawbacks to utilising the MRI/PET approach. The first pertains to the difficulty and cost associated with producing and transporting the radiopharmaceuticals required for PET imaging [10]. The half-life of radioactive fluorine, the chemical used to trace glucose metabolism, is merely 2 h [33]. Its production is not only expensive, but can produce false negative and positive results, meaning the investigation is still considered to be under continuing research [29,30]. Second, although more accurate pre-operative imaging may assist in the determination of the tumour border, translating an image into surgical margins will always prove to be difficult. 

## 3. Intra-Operative Imaging

Image guidance during OS resection may aid in the accuracy of identifying the tumour edge. Surgeries involving OS may implement fluoroscopy, an imaging technique widely utilised throughout the orthopaedic field [34]. However, not only does its use entail radiation exposure to both the patients and medical staff, [34] the primary issue described above is not addressed: tumour margins are still difficult to appreciate, as they are not easily discernible through fluoroscopy alone [4].

**Figure 2:**
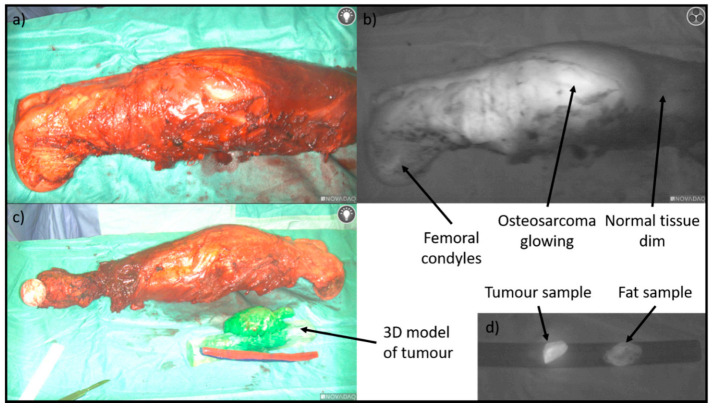
(**a**) Shows the resected femur and OS contained within vastus medialis. (**b**) Shows the specimen through the infrared camera (Stryker), with the OS glowing bright. (**c**) Shows the resected specimen next to the three-dimensional (3D) printed model produced prior to the procedure (Axial3D). (**d**) A sample of the tumour was dissected out of the specimen to demonstrate higher fluorescence compared to a piece of fat.

The majority of image-guided cancer resections utilise optical imaging techniques such as near infrared camera systems [35,36]. However, there are currently only a handful of approved fluorescent agents that are available, primarily fluorescein and indocyanine green [37,38]; the use of the latter has been described in OS [39] (Figure 2 shows its use in OS). Although these have shown some promise, these agents are not specific to sarcoma and therefore research is ongoing into the development of monoclonal antibodies conjugated with infrared dyes which can bind to sarcoma cell surface targets [35].

**Figure 3:**
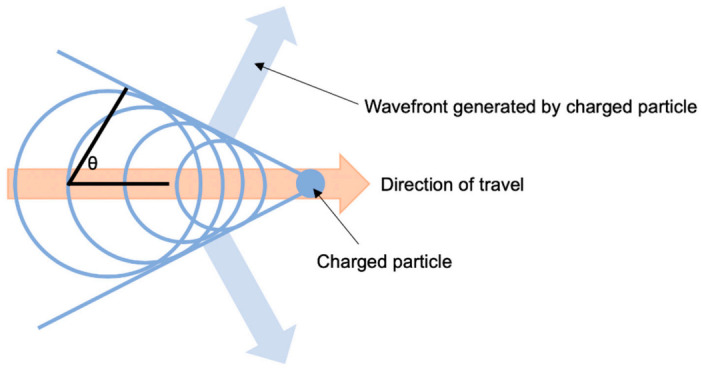
Huygen’s construction of a conical Cherenkov wavefront-a charged particle traveling in a given direction transmits its kinetic energy to the surrounding media, depicted by the larger circles trailing behind the particle. Cherenkov radiation is generated at an angle to the direction of the travelling particle, defined as θ, which is related to the energy of particle [35]. Therefore, the higher the kinetic energy of the particle, the wider the generated wavefront, and hence the more easily the radiation can be detected.

Cherenkov luminescence (CL) describes an imaging technique in which radionucleotides accumulate in a tumour and decay, emitting charged particles [35,40] (Figure 3). These charged particles result in the emission of photons from surrounding dipoles which can be detected using infra-red cameras [41,42]. CL imaging is beginning to show increasing value, primarily in preclinical trials [43,44]. Several medical isotopes have been shown to be clinically relevant, including ^18^F, ^15^O, ^13^N, ^68^Ga, ^89^Zr, ^64^Cu, ^225^Ac, ^90^Y, ^124^I, and ^74^As [35]; these isotopes are then conjugated to compounds such as monoclonal antibodies that accumulate in or around the tumour cells. The specificity of these isotopes can be further improved via conjugation to molecules specific to receptors on the tumour, such as HER-2 antibody Pertuzumab [45] or prostate specific membrane antigen (PSMA) [46,47]. Additionally, the development of novel optical agents is not required, as CL imaging takes advantage of already approved radiopharmaceuticals [40]; many of these, such as ^18^F-FDG [48] and ^68^Ga-PSMA [46,47], are also used as radiotracers for PET imaging, making them ideal bimodal agents.

The resolution for CL imaging has been shown to be far better than any concurrent nuclear imaging modality [49,50], being able to identify much smaller structures than PET scanners, which are currently deemed gold standard [35]. Despite their high resolution, the average amount of CL produced is rather low [51], necessitating highly sensitive instrumentation for its detection, alongside longer imaging times of several minutes [35]. Fortunately, such camera systems are already available, currently being used primarily for chemiluminescence and bioluminescence imaging [52]. 

Unfortunately, there is limited data on the use of Cherenkov radiation for intraoperative margin assessment in human studies, with none in OS, although small scale feasibility studies have been published for breast [48] and prostate cancer [46,47]. All studies demonstrated promising results, with CL imaging assessment of margins generally correlating with histopathological assessment. A further drawback of CL imaging is that some of the light emitted from the radionucleotides is absorbed by the surrounding tissue [35,53], posing a limitation for deep tissue imaging. In the human studies published so far, CL imaging was not conducted whilst the tumour was in situ, rather it was conducted after the tumour has been excised [46,48]. This could avoid the issue of depth in OS but is less preferable to being able to assess margins prior to excision, as it does not reduce the risk of taking too much tissue. 

Therefore, CL imaging may be able to provide a new imaging modality that utilises existing clinical radioactive tracers with concurrent optical imaging technologies for intraoperative imaging, merging nuclear and optical imaging [35]. Given the use of ^18^F-FDG as a radioactive tracer for pre-operative PET in OS is established [32], the use of CL in OS surgery is a promising avenue.

## 4. Computer-Assisted Navigation

**Figure 4:**
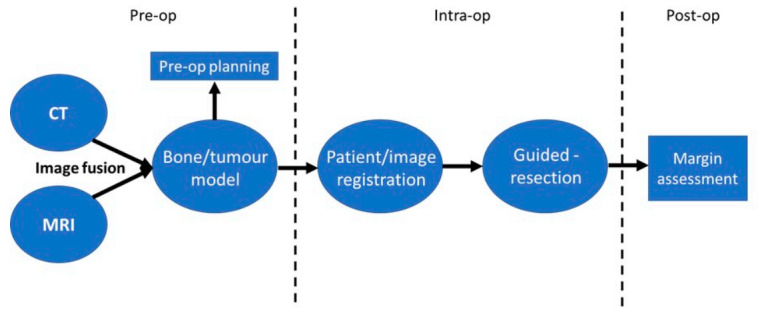
CANS flowchart: adapted from Wong et al. [56].

Computer-Assisted Navigational Surgery (CANS) is another form of intra-operative guidance establishing its usefulness in OS for both joint-preserving surgery, and pelvic tumour resection, both of which require complex and precise osteotomies [36,54,55]. CANS begins pre-operatively with multimodal image fusion; CT images providing good bony detail are combined with MRI images detailing the tumour extent to form a three-dimensional (3D) “bone-tumour model”, allowing the surgeon to plan their margins and reconstruction pre-operatively [55]. Intra-operatively, the image is then registered to the patient via the placement of trackers on anatomical landmarks and calibration to the image with the navigation probe [56]; image-patient registration must then be assessed to confirm accuracy (Figure 4). The navigation probes can now be visualised in real-time on the bone tumour model to identify one’s position relative to the tumour and facilitate execution of the pre-planned cuts.

### 4.1. Joint Preserving Surgery (JPS)

Although limb salvage surgery is now preferable to amputation, it is still not without its flaws. Replacement with endoprostheses bears many of the complications similar to regular arthroplasty, such as aseptic loosening and infection, with high rates of both structural and soft tissue failure [57,58]. Given that OS commonly arises in adolescents [2], this is problematic as they will be required to stay *in situ* for many years. Furthermore, although some implants can be lengthened by a reasonable amount, limb length discrepancies and joint dysplasia are significant long term issues [58].

Joint Preserving Surgery (JPS) aims to reduce the above complications by preserving the patient’s native articular surfaces and ligaments [1], and has been shown to potentially improve post-operative joint mobility [59]. JPS has been shown to preserve future limb growth if the physis is spared when used for OS arising around the knee [60], so is particularly useful in skeletally immature patients. JPS is not possible in all patients; indicators of its suitability are good response to neoadjuvant chemotherapy, achievable margins of ≥10 mm, no unresectable metastases and residual epiphysis of >10 mm [1,61]. Whilst no physeal extension of the tumour is preferable, both in terms of ease of resection and future growth, it is not a necessity [1]. In order to preserve the physis whilst maintaining sufficient margins, precise osteotomies and careful planning are pre-requisites, both of which can be aided with the use of CANS [62].

It was first described by Wong et al. in 2008, in which CANS was used to perform four JPS procedures [63]. Three of these patients required extremely accurate resections given the pre-planned cuts would only leave 1.5–2 cm of epiphysis; the authors felt that CANS helped them to achieve this [63]. The mean follow up in this study was only 9.3 months so interpretation of recurrence/survival data is difficult [63]. In 2013 they published a second paper, in which they described JPS using CANS for eight patients, six of whom had OS, this time with a mean follow up of 41 months [61]. All margins were within 2 mm of that planned and patients had a mean Musculoskeletal Tumour Society (MSTS) score of 29/30, suggesting good functional outcomes were achieved. Furthermore, there were no local recurrences within this time [61]. Li et al. published two similar studies, the first in 2012 in which clear margins were achieved in all six periarticular OS and an average MSTS of 26.6 was achieved [62]. Their second study included seven peri-articular OS, this time followed up for a mean of 25.2 months; again, clear margins were achieved in all and a mean MSTS of 26.3 was achieved [64]. There was no local recurrence during this time [64]. Both authors felt that CANS aided them in their ability to accurately perform the planned resection and achieve the desired margins. 

### 4.2. Pelvic OS Surgery

**Figure 5:**
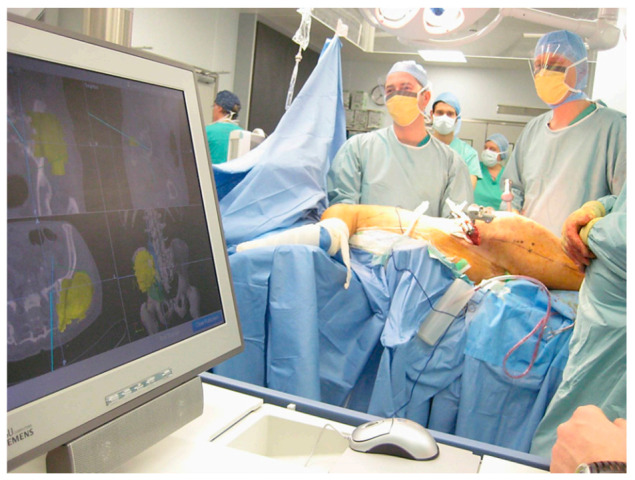
CANS in use for a pelvic tumour resection. The screen on the left shows the bone-tumour model with the position of the navigation probe superimposed.

Pelvic tumour resection also requires precise and difficult osteotomies, owing to the complex anatomy of the area [11,12,13]; this may contribute to a pelvic tumour’s association with increased local recurrence and poorer prognosis [14,65]. Cartiaux et al. asked four established tumour surgeons attempt to resect simulated tumours from pelvic models; they found there was only a 52% chance that the planned margin of 10 mm (+/−5 mm) was achieved and that the reconstructions achieved were generally poor [66], before showing a reduction in error using CANS technology on the same models in a second study [67]. Figure 5 shows an example of the technology in use for pelvic tumour resection.

Both Hüfner and Krettek published papers in 2004 demonstrating the feasibility of CANS for pelvic sarcoma surgery, achieving clear histological margins in all patients (*n* = 3 and *n* = 2 respectively) [68,69]; neither study included OS patients however. Cho et al. further evaluated the use of CANS in pelvic tumour surgery; of the 10 patients included, all had clear margins and only two developed local recurrence after a minimum of three years follow up, with the authors feeling that it increased the accuracy of resection and minimised the resection of unnecessary healthy tissue [70]. This study included two OS, one pelvic and one sacral; the pelvic OS patient remained disease free at 38 months whilst the sacral OS patient passed away 22 months later after distal recurrence of disease [70]. Results from Wong et al. echoed this; all resections were within 2 mm of the planned margin, with 3 out of 12 patients with pelvic/sacral tumours having local recurrence after a minimum of 3 years follow up [71]. Again, this paper included one pelvic OS and one sacral OS, with the pelvic tumour remaining disease free at 46 months, whilst the sacral tumour died at 22 months [71].

Whilst these papers demonstrate the feasibility of CANS for pelvic OS, the benefits have perhaps been overstated in previous reviews, such as one by Wong himself [56]. The local recurrence rates from these (25% for Wong [71], 20% for Cho [70]) papers are directly compared to Ozaki’s loss of local control rate for standard treatment (70%) [65]. Out of the 67 of patients involved in Ozaki’s study, 17 received no definitive surgery; the recurrence rate was 62% for those that did. Furthermore, Ozaki’s study contained only high-grade OS, with 34/67 having metastases at diagnosis [65], so have a worse prognosis from the outset than these mixed cohorts of sarcomas; the presence of metastases was a specific exclusion criteria in the paper by Cho et al. [70]. The local recurrence rate was only 27% in a retrospective analysis of 539 primary pelvic bone tumours treated with standard surgery by Jeys et al. [72].

In the absence of prospective comparative studies, it is difficult to say that CANS reduces local recurrence rates for pelvic tumours, especially for OS. What is perhaps more relatable to OS is the ability to achieve a more reliable margin using CANS for pelvic tumours, given that the margin is closely related to prognosis in OS [11,12,13] and this increased accuracy likely applies to all tumour types. Jeys et al. operated on 31 pelvic tumours, including three OS, using CANS and found that they had a positive margin rate of 8.7%, markedly lower than their previously published rate of 29% when using a standard surgical technique on 539 primary pelvic bone tumours [73]. Whilst there is no randomisation or case matching here, when combined with previous reports, this would suggest that CANS is likely beneficial for achieving an accurate resection margin in pelvic OS. Furthermore, Laitinen et al.’s case comparison study (containing 10 OS out of 21 pelvic tumours) suggested that CANS was safer, reducing post-operative foot drop, blood loss and the total operating time, although these did not reach statistical significance [74]. Significance was reached however with regards to increased disease-free survival in the CANS cohort; this should be interpreted with caution however given the far shorter mean follow up in this group (23.2 months vs. 60.7 months) [74].

### 4.3. Limitations and Future

There are a number of limitations with the technology at present. Firstly, there is a lack of good evidence confirming its efficacy: all studies contain small sample sizes and are non-randomised, retrospective studies. The cost of the technology must also be considered, as well the fact that it only aids in resection of bone tumours, and not soft-tissue tumours. The accuracy of CANS relies on the registration process which is user dependent. 

One of the limitations of this technology is the lack of saws or osteotomes [56] which function as navigational probes and are visible on the imaging; this would allow real-time representation of cuts made on the patient imaging, allowing adjustments to be immediately to ensure execution of the planned margin. Whilst its use in tumour surgery has not yet been published, the Mako robot (Stryker) could be of use here. The Mako robot-assisted system is effective for uni-compartmental and total knee replacement, in which precise prosthesis placement is crucial to ensure a good outcome [75]. This would allow the surgeon to track the cuts in real time, whilst the robotic arm aids steadiness and helps one remain in the desired plane.

## 5. Three-Dimensional (3D) Printing

Three-Dimensional (3D) printing has revolutionised modern day manufacturing of geometrically complex, unique and one-off models and products. Although there are now many different types of 3D printing techniques with differing complexities, the basic premise has remained and involves 3D objects being created by the addition of material layer by layer [76]. Whilst the technology was initially developed for use within the engineering and industrial sectors, 3D printing has quickly evolved and is now adopted in almost all sectors of society and is even becoming a household item [77]. This technology has been rapidly adopted by the medical field and has been particularly desirable within surgical specialities, since it has allowed surgeons to visualise, hold and even practise the operative approach for complex operations, facilitating a personalised approach to modern-day surgery [78]. Amongst these, orthopaedics, maxillofacial and oncological specialties are some of the biggest implementers of the technology, given the prevalent use of biomaterials and mechanical implants [79]. Since OS surgery combines orthopaedics with tumour resection surgery, 3D printing has become an important tool for aiding the surgeon and optimising patient care.

The basic workflow for how 3D printing can be used to aid in the management of OS involves a number of steps [77]:

1. Common imaging modalities that are used during the diagnostic and pre-surgical workup, such as CT and MRI, are converted into a 3D reconstruction of the desired anatomical structure.

2. The next step depends on the desired use of the 3D printed model:

(a) The 3D reconstruction can be directly converted into a .STL file and printed via the desired additive manufacturing method. This provides a 3D replica which can be used for visual pre-surgical planning, testing the suitability of an implant or device, and in some cases for patient education during the consent process. This is known as the indirect technique.

(b) Today, more commonly, the 3D reconstruction can be manipulated using a computer aided design software (CAD), often under the guidance of an engineer. Instead of simply creating a 3D replica, this allows surgeons to plan operations virtually and create patient specific implants (PSI) (discussed in Section 6), cutting guides and drilling paths which perfectly match the anatomical area. These designs can then be manufactured by 3D printing. Often, a low-cost prototype is printed first to check suitability before the sterile PSI or guide is manufactured in the chosen material. This is known as the direct approach. The common materials used in 3D printing are described in Table 2.

### 5.1. D Printed Cutting Templates

**Figure 6:**
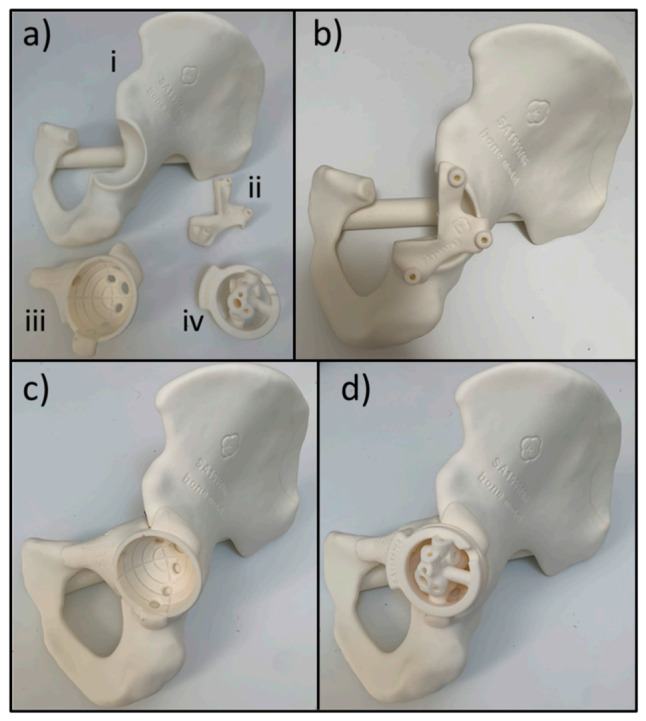
Images demonstrate the 3D printed components used for the accurate resection of a pelvic tumour. (**a**) shows 3D printed components involved—(**i**) is a 3D printed model of the patient’s pelvis post-osteotomies, (**ii**) is the guide for the posterior and inferior cuts, (**iii**) is a template of the 3D printed implant, (**iv**) is the drill bit guide jig. The superior pubic ramus was cut under computer assisted navigation surgery (CANS) guidance, before the posterior and inferior cut saw guide was positioned as shown in (**b**) and held in place with pins. After the cuts were made, the template was positioned in the patient as shown in (**c**). The drill bit guide was the positioned as shown in (**d**) which, in conjunction with the implant template, ensured the screw holes are drilled correctly for the custom implant. Implants from Implantcast GmbH.

For OS resection surgery, the direct approach offers a significant advantage over traditional surgical management. One of the most established and versatile applications of 3D printing within OS surgery involves custom designed cutting templates [89], demonstrated in Figure 6. This allows the surgeon to execute resections accurately following pre-operative planning, to ensure adequate tumour margins without resecting excess tissue which increases patient morbidity and delays the recovery process. However, the associated costs, time to set-up, requirement of precise registration and extensive training limits its accessibility and wide implementation. A study by Ma et al. showed that 3D printed guiding templates led to more precise tumour resection, less blood loss and shorter operative time compared to traditional surgical techniques, whilst being cost-efficient [89]. Furthermore, a cadaveric study by Wong et al. showed similar resection accuracy and decreased resection time when using PSI cutting guides as opposed to CANS for simulated pelvic tumour surgery [90]. Another use of the direct approach involves CAD design of custom drilling guides to ensure secure implant fixation within viable bone, which has been previously reported in OS surgery [91]. This is especially valuable for thin bone sections, such as the pelvic ischium and periacetabular region, and aids the surgeon to obtain adequate screw purchase. The accuracy of this technique however relies entirely on accurate placement of the template; there is no registration process akin to that used in CANS to confirm correct placement of the device [56].

### 5.2. 3D Printed Tumour Models

**Figure 7:**
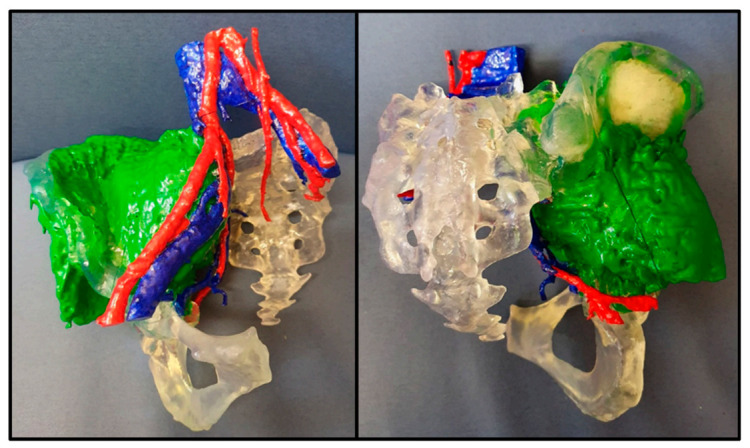
Images show a 3D printed model of a pelvic tumour with a large soft tissue component, demonstrating both the extent of the tumour, and its relationship to the blood vessels (model from Axial3D).

The indirect approach also has benefits for OS surgery. By 3D printing the tumour (Figure 7), it can help the surgeon and theatre staff to orientate themselves before and during the operation, which could reduce the operative time and thus costs. It has already been shown that for maxillofacial and orthopaedic specialties, 3D printed models can save time in the operating room [92]. The wider use of 3D printing in the management of OS should also be mentioned. Notably, 3D printed tumour models can be used to better educate and consent patients in pre-operative clinics as previously shown in other specialties [93], and can help to promote Patient and Public Involvement for research purposes, which could increase participation to future trials.

## 6. Implant Advances

Effective reconstruction aims to restore the patient’s functionality to a high standard whilst minimising complications and the need for subsequent revision surgery. Options include implantation of metallic endoprostheses and biological reconstructions using allografts or autografts [94]. Numerous studies have demonstrated that implants provide satisfactory functional outcomes including the rapid return to weight-bearing [95], making them the preferred choice of reconstruction across the majority of centres [96]. Although complications that hinder implant survivorship such as mechanical failure, aseptic loosening and infection pose challenges, advances in implant technology look to overcome them.

### 6.1. Modular Implants

Traditionally, implants were made on a case-by-case basis by manufacturers in a costly and time-consuming process. Depending on the complexity of reconstruction, these so called ‘custom’ implants took 4-12 weeks to be manufactured [95]. In contrast, modular implant systems are ready to use off-the-shelf, decreasing the time between diagnosis and surgery. Additionally, they are less expensive and show good survivorship [97]. Another major advantage is the greater flexibility they offer. Surgeons are able to combine several different components together intraoperatively to form an implant that best matches the patient’s bone defect [95]. As well as the components being standardised for improved quality control [98], the versatility allows modular systems to be utilised for total bone replacements in addition to segmental reconstructions [99]. As a result, for many cases, custom implants are not required and modular systems are effective. Schwartz et al. retrospectively reviewed 186 patients, primarily with OS, by comparing 85 patients treated with modular implants versus 101 with custom implants and found a 15-year implant survivorship of 93.7% and 51.7%, respectively [100]. A study by Gosheger et al. which included 139 OS patients treated with the MUTARS® implant (Implantcast, Buxtehude, Germany), revealed a 5-year patient survival rate of 70.4% [101]. Since a significant portion of OS patients are young people, greater demands are placed on the implants as many are expected to lead active lives [102]. Lang et al. demonstrated that both prior to diagnosis and 5 years following implantation of a modular endoprosthesis, 24 out of 27 OS patients were able to play sports [103].

### 6.2. Extendible Implants

Leg length discrepancy (LLD) is an issue arising from the resection of affected growth plates. Whilst JPS can avoid this [60], it is often not suitable, as mentioned above. An increasing body of evidence suggests extendible implants provide good compensation for LLD. Modern versions can be elongated non-invasively using a magnetic force, aiming to decrease the number of operations and associated anaesthetic and infection risks [104]. Their use, however, is not yet widespread due to complications. Cipriano et al. treated 10 patients using the Repiphysis Limb Salvage System (Wright Medical Technology) and observed 15 reoperations for 37 implant-related complications, notably aseptic loosening [105]. Yet, Gilg et al. used the custom-made Juvenile Tumour System (Stanmore Implants Worldwide, Borehamwood, UK) on 50 OS patients and discovered a 5-year revision-free survival of 61.6% and an average limb elongation of 39 mm [104]. Similarly, Torner et al. achieved a mean lengthening of 36.4 mm using the MUTARS® Xpand Growing Prosthesis (Implantcast, Buxtehude, Germany) [106]. Zou et al. found the LLD was ≤2 cm in 20 out of 33 patients [107].

### 6.3. 3D printed Implants

**Figure 8:**
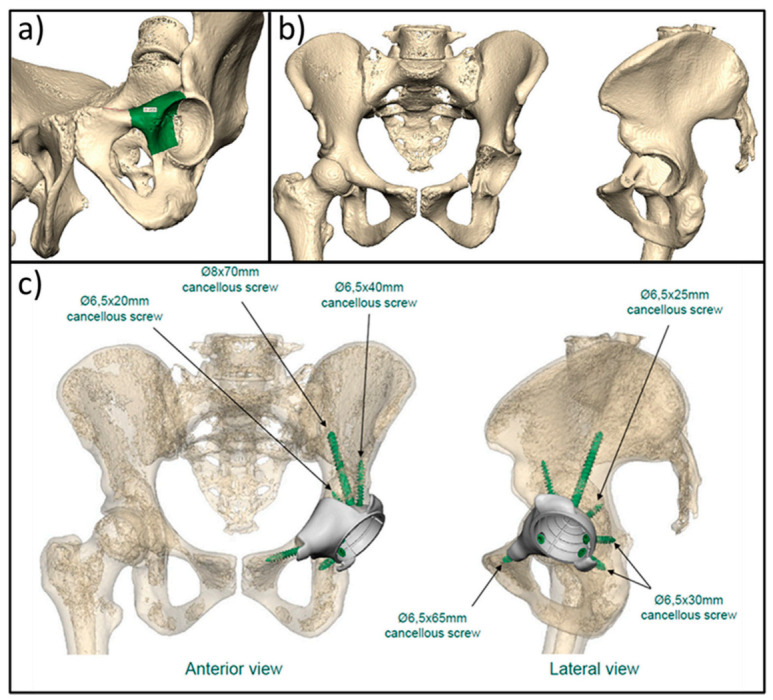
Images show the planning stages of a 3D implant. (**a**) Shows the identification of the margins required on a 3D image of the patient’s pelvis, whilst (**b**) Shows an image of the patient’s pelvis post resection. This identifies the deficit that needs to be replaced, which can then be designed, as shown in (**c**).

Developments in 3D printing has facilitated the use of a wider range of materials and has taken the technology beyond prototyping [108]. Although modular implants have shown good results, when the tumour is large or involves complex anatomy, a 3D printed PSI can offer a superior fit [109]. Pre-operative imaging can be processed to design custom implants which can now be printed layer-by-layer, enabling the construction of more complex geometries as a single unit [95] (Figure 8). This is particularly useful when the patient’s anatomy is considered unsuitable for modular implants [99]. Moreover, 3D printing is less time consuming and more cost efficient in comparison to conventional methods of custom implant manufacturing [108]. Although the evidence base is limited, especially regarding long term outcomes, promising results are being found. Liang et al. performed pelvic en bloc resection and used a custom 3D-printed titanium alloy implant on 35 patients, including 11 with OS. At a mean follow-up of 20.5 months, no deep infection or loosening was discovered. They attributed this to a shorter operating time and the accurate matching of contours between the resection plane and implant [110]. Comparable findings were observed by Hu et al. who performed reverse shoulder arthroplasty on seven patients (three with OS) using a custom 3D-printed glenoid implant which took only 7–10 days to produce. Again, no deep infection or loosening was reported over an average follow-up duration of 23.6 months [111]. Liu et al. obtained similar results and accredited this to the porous surface of their implant which was achieved via 3D printing [112]. The benefits of surface porosity on the promotion of osteointegration is well accepted [113].

A further advantage of creating PSIs using 3D printing is the choice of material, which can be tailored to the application and patient’s needs. This is an evolving area, however some groups have shown progress with biomaterials such as polyetheretherketone (PEEK) using state-of-the-art printing techniques [114]. Whilst off-the-shelf implants are still widely used for reconstruction in OS surgery, PSI technology is improving and offers clear benefits for both surgeon and patient. As the costs of 3D printing fall further, this may become a more readily available option for more patients with OS to enhance outcomes. As 3D implants are produced prior to surgery, precise resections are required to ensure good implant fit; something which CANS has be found to be effective for [71]. The ‘Just in Time’ project by ACMD is currently in its early stages, but aims to take these technologies further, combining CANS techniques described in Section 4 with new lattice structure 3D printing techniques to improve both implant quality, and the speed at which they are available [115]. Their goal is that, in the future, these implants can be printed intra-operatively, in real time.

### 6.4. Implant Coatings

Infection is the principal cause of implant failure [98]. It can lead to loss of bone stock which often necessitates amputation [116]. Infection risk is particularly high in OS patients due to long operating times, extensive dissections, and chemotherapy/radiotherapy use [95]. Due to their non-biological composition and dead spaces, bacteria can adhere to implants and form biofilms. However, implant coatings help combat this. Silver is well known for its broad-spectrum antimicrobial properties, but there has been concern about potential toxicological side effects [117]. Nevertheless, in a study comprising 98 patients, predominantly with OS, Hardes et al. exhibited lower infection rates in patients with a silver-coated titanium megaprosthesis (8.9%) compared to uncoated (16.7%) after a median follow-up of 8.2 years [118]. The coated group also showed a better 5-year survival rate (90% versus 84%) and no local or systemic side effects were observed [118]. Hussmann et al. also found fewer infections with silver-coated implants compared to uncoated (5.6% versus 22%), along with a shorter duration of hospital stay [119]. Silver coatings are now applied to systems offered by a number of implant manufacturers including Implantcast who coat their MUTARS^®^ components through electroplating and Stanmore Implants Worldwide who use Alguna^®^ by Accentus Medical (Didcot, UK) [117].

Iodine coatings have also shown promising short-term results against infection [120,121] but there is a lack of comparative studies and evidence for its use in OS patients. Numerous other compounds for anti-bacterial implant coatings are emerging with many still in their preclinical phase [122,123,124].

### 6.5. 3D Printed Drug Delivery Systems

Finally, 3D printed biodegradable implants as a drug delivery system have been reported. Wang et al. have described a 3D printed poly L-lactic acid (PLLA) implant as a localised chemotherapy delivery system for OS [125]. This method involves 3D printing, drug loading, drying and implantation [125]. Whilst this is still an early concept, the mice models in this study showed a very high local drug concentration with sustained duration, both of which increase cytotoxicity at the tumour site. This novel adaptation of 3D printing could improve OS outcomes due to localised and individual pharmacotherapy, whilst reducing systemic effects of chemotherapy agents. However, the efficacy of this technique is yet to be tested in humans and is likely to be dependent and limited by the chemotherapy agent used. Furthermore, a key aim of adjuvant chemotherapy is to improve cure rates by eliminating covert metastases, which relies upon systemic administration [126]. Nevertheless, the option to localise cytotoxic agents using 3D printed implants is a unique and promising avenue for the future of OS management.

In addition to cytotoxics, 3D implants could also be combined with growth factors and stem-cells in order to better stimulate vascularisation and osseointegration of the implants. To our knowledge, this has not yet been used in OS, but primitive forms, such as the Infuse Bone Graft, have been used in spinal surgery [127]. These consist of collagen sponges laced with bone morphogenic protein 2 (BMP-2), but unfortunately have been associated with high levels of complications, such as heterotopic ossification and various neurological complications [128,129]. Experimental work has shown that improved bioprinting techniques, in which the distribution and timed release of growth factors BMP-2 and vascular endothelial growth factor (VEGF) reduce heterotrophic ossification and enhance bone defect healing in a mouse model [127]. Experimental work is also under way to create 3D printed implants containing osteogenic cells; this has proved problematic given the difficulties in producing materials that are non-immunogenic and have suitable porosity to allow angiogenesis and osseointegration, whilst maintaining suitable mechanical strength for load bearing [130,131]. The hope is that these implants will eventually reduce complications such as aseptic loosening.

## 7. Biological Reconstruction

Whilst implant technology has improved, implants still have many flaws as discussed above. Sometimes it is necessary to reconstruct the defect with biological tissue, often in procedures such as JPS (described in Section 4.1). There are 2 main established techniques: allografts and autografts, with combinations of the above providing advancement in reconstruction.

### 7.1. Allografts

Bone allografts describe the implantation of bone donated from a third party, with the aim of integration with host bone [1]. These can be either non-structural or structural; the former often describes chipped bone used to replace a deficit in bone, often after techniques such as curettage, whilst structural grafts are load-bearing and more commonly used to replace intercalary sections of bone post-resection [132]. Whilst allografts are common practice and well established, they have previously been associated with high rates of complications, principally non-union, infection and pathological fracture [132,133,134]. Most studies had only short follow up times and used extrapolation or surrogate markers to determine long-term outcomes.

More recently, a paper published by Sanders et al. evaluated the long-term outcomes of allografts for intercalary reconstructions. A total of131 patients (55% OS) were followed for up to minimum of 10 years; infection rates were minimal, but 16% experienced non-union, whilst 19% suffered allograft fractures [135]. Interestingly, fixation with intra-medullary nail only and fixation non-bridging plates were associated with an increased rate of fracture. Given the complication rates were high, they felt the main reason it was an acceptable method of reconstruction was the lack of alternatives [135]. A similar study by Aponte-Tinao et al. also evaluated outcomes for 193 patients (63% OS) over a 10 year period for large allografts. Like the previously mentioned paper, they found similarly high rates of fracture and non-union, but also found an infection rate of 14% [136]. Overall, they found that after 10 years there was a 40% risk of allograft removal, joint replacement, or amputation, with the risk highest for osteoarticular tibial grafts [136]. These studies identified less than desirable outcomes from these allografts but identified prognostic factors which could help better determine their suitability in the future. They provide reasonable structural strength but are let down by their high non-union and infection rates. They are further hampered by their economic cost and often problematic availability [137].

### 7.2. Autografts

Bone autografts describe the implantation of the patient’s own bone tissue when reconstructing the resection defect. The main advantage is that the bone segment that has been resected will obviously exactly match the defect for reconstruction. Broadly, these autografts fit into 2 main categories: tumour devitalised autografts and free vascularised fibula grafts (FVFG). Tumour devitalised autografts involve the reimplantation of tumour bearing bone tissue, after devitalisation, to fill the resection defect [1]. A number of different methods have been described for the devitalisation of grafts prior to re-implantation, mainly forms of heating/cooling or radiation. 

Devitalisation via heat can be achieved with pasteurisation [138,139,140]. This was first described by Manabe et al. who resected the tumour containing bone and placed it in 60° saline for 30 minutes to devitalise the tumour, prior to submersion in room temperature saline, before finally re-implanting the graft [138]. This included 25 cases (13 OS), with a non-union rate of 23%. Qu et al. assessed patients treated with this approach and found excellent functional outcomes with a mean MSTS of 93%, although with a mean follow up of 11 months, this does not provide much information about long term outcomes. This was better assessed by Jeon et al. who followed-up patients for an average of 74.3 months—Kaplan Meier analysis found a 10 year graft survival rate of 74% [139]. They felt it to be a more accessible and economic alternative to allograft with similar outcomes. An alternative to pasteurisation for devitalisation is liquid nitrogen freezing, for which there are 2 main methods: free freezing and pedicle freezing [1]. Both techniques involve submersion of the tumour in liquid nitrogen after curettage of intramedullary tumour; free freezing describes resection of the tumour prior to submersion whereas in pedicle freezing, the tumour is not resected from the long bone, but the bone dissected out and submerged whilst attached [141]. A comparative study suggested pedicle freezing achieved faster bone-graft union and a lower complication rate [142]. The long-term outcomes of 72 patients (32 OS) treated with frozen autografts were reported by Igarashi et al. who found an autograft survival rate of 80.6% at 10 years, with excellent functional outcome in 72.2% of patients [143]. It has been suggested that freezing is a more effective method of preparing tumour devitalised grafts compared to pasteurisation, as it better preserves the osteoinductive ability of the graft [144].

The other major method of devitalisation is that of extra-corporeal irradiation (ECI); this was first described in 1968 [145] and has since become well established. Here, the tumour is resected prior to ex-vivo radiation (~50 Gy) and re-implantation with either an intra-medullary nail or intra-medullary cementing. Puri et al. recently reported a series of 70 diaphyseal sarcomas (38 OS) with a minimum follow up of 3 years; there was a non-union rate of 36.5%, with a 5 year implant survival rate of 79% [146]. The infection rate in this study was 12% whilst fracture rate was 6%. Interestingly, non-union was significantly more likely to occur in diaphyseal osteotomies than metaphyseal osteotomies, whilst all local reoccurrences occurred in the soft tissues, not the graft, suggesting the technique is oncologically safe [146]. A prospective randomised study by Wu et al. compared ECI with frozen-autografts for OS. They found no difference in fracture, infection or non-union rates, and ultimately 5-year survival, between the two groups [147]. Considering the above information, it appears that tumour-devitalised grafts have a similar level of effectiveness and complications as allografts, but with the added benefits of being cheaper and more readily available.

FVFG are also an established form of autograft used in OS reconstruction surgery. They carry several benefits over devitalised autografts, largely owing to their intact vascular supply and the fact that it is living tissue. This allows the graft to continue to hypertrophy after implantation, whilst aiding union and providing resistance to infection [148]. Eward et al. evaluated the use of FVFG for large skeletal defects in tumour surgery (*n* = 30, 14 OS), and found that although FVFG achieved good rates of union, fracture rates were high at 20% [149]. In comparison to allografts and devitalised autografts, FVFG appears to have increased oncogenic ability, aiding union, and a reduced risk of infection, but decreased strength posing an increased risk of fracture.

### 7.3. Graft Combinations

The above techniques do not necessarily exist in unison; in as early as 1993 the so-called Capanna technique combined allografts with a FVFG [150]. This aimed to combine the structural properties of allografts whilst reducing the non-union rates via utilisation of the vascular and osteogenic properties of the vascularised free fibular grafts [151]. Capanna et al. later reported the results of 90 patients followed up for a mean of 9 years, describing a 93% success rate, with non-union and fracture rates of 8.8% and 13.3% respectively [152]. This seems like an improvement on allograft alone, although there are no comparative studies to our knowledge at this time. Multiple groups have utilised and reported this technique [153,154].

Whilst the traditional Capanna technique combines allograft with the FVFG, Lu et al. recently combined it with frozen tumour-bearing autograft for lower limb OS, combatting the issues related to limited availability of suitable allografts [137]. This study compared the new method directly against the Capanna method for 23 patients (*n* = 8 and *n* = 15 respectively) and found a significant reduction in mean time to union (8.4 months vs. 14.1 months) using autograft, whilst maintaining similar functional and oncological outcomes [137]. Whilst such results are promising, the study is small-sized, retrospective and non-randomised; therefore, further work is needed to assess its efficacy. The addition of FVFG has also been shown to improve repair at a histological level when combined with pasteurised allografts [155]. Hong et al have also described the combination irradiated autografts with FVFG, but they did not report their results separately to those without concurrent FVFG [156].

## 8. Relevance to Head and Neck Surgery

OS in the head and neck is extremely rare. It accounts for <2% of all sarcomas occurring in the head and neck [157]. When it does occur, the mandible is the most common site, followed by the maxilla [158,159]. Surgical resection remains the mainstay of treatment with curative intent. The role of adjuvant and neo-adjuvant chemotherapy in patients with adequate surgical resection has been unclear [160] but there is some evidence that neo-adjuvant chemotherapy may provide a survival benefit and lower local recurrence rates [161,162]. Five year survival rates for OS in the head and neck have been shown to be 50–60% [159,163] with better survival noted in paediatric populations [164]. Surgical margin status has been shown to be the most significant prognostic indicator followed by tumour grade [163].

The anatomical constraints of the head and neck due to the high density of functionally and cosmetically sensitive structures can make wide resection of sarcomas challenging. Despite this, aggressive early resection and appropriate reconstruction undertaken within a specialist multi-disciplinary team setting including adjuvant chemo/radiotherapy can give good oncological and functional outcomes [157,164].

**Figure 9:**
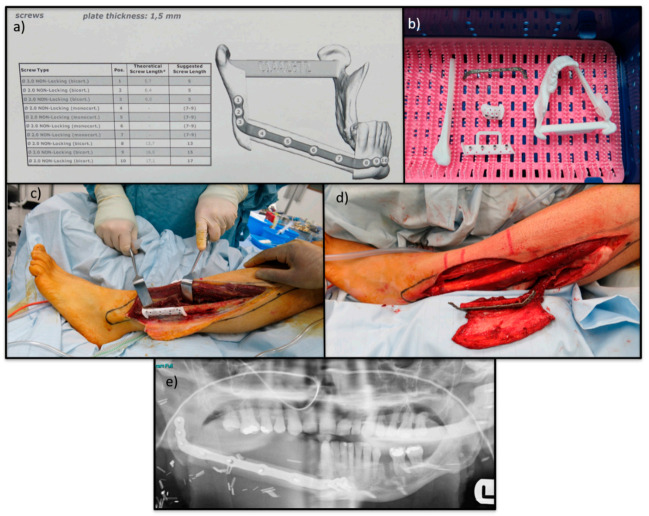
Images show the reconstruction of a mandibular OS. (**a**) Shows the reconstruction plan including the pre-determined screw lengths and locations. (**b**) Shows the 3D cutting guides, 3D model and the custom reconstruction plate. (**c**) Shows custom cutting guide on free fibula osseocutaneous flap. (**d**) custom reconstruction plate attached to free fibular osseocutaneous flap. (**e**) Shows the follow up orthopantomogram with free fibula osseocutaneous flap fixed in defect.

Reconstruction of the mandible and maxilla following wide resection is best performed with vascularised bone [165]. Microsurgical reconstruction of these defects with free osseo-cutaneous/osseo-muscular flaps is the accepted gold standard [166]. The most recent surgical advance includes 3D planning and printing of resection guides and reconstruction plates which allows for customisation and provides optimum oncological and reconstructive surgery with reduced operating times [167](see Figure 9).

## 9. Relevance to Spinal Surgery

Spinal OS is rare accounting for less than 5% of malignant tumours of the spine [168]. They are aggressive with an overall median survival of 6.7 years [169]. The vital anatomical structures of the axial spine mean that unlike limb OS, wide excision is rarely possible without significant damage to major blood vessels or the spinal cord. As such marginal excision is usually the surgical goal. Surgical excisions are termed Enneking appropriate (for en-bloc resection with marginal margin) or Enneking inappropriate (positive margin at histology). Reduced local recurrence and longer disease-free survival is achieved with Enneking appropriate resections [168,169].

Recent advances in spinal tumour surgery include implant materials, access techniques and navigation. 

### 9.1. Implant Materials

Modern metallic spinal implants are strong, intuitive and modifiable for any area of the axial skeleton; however, as with limb surgery, they can interfere with modern imaging for detailed surveillance or advanced radiotherapy techniques. As a result, the use of PEEK cages and in particular carbon fibre implants are becoming more common place. Strength, stiffness and fatiguability appear equivalent or superior to metallic implants. Costs can be high however and with an inability to contour rods they are less user friendly, however the surveillance imaging and subsequent radiotherapy planning is far superior [170].

### 9.2. Access Techniques

Because of the confined spaces of the thorax, retroperitoneum or pelvis as well as the muscle damage caused by conventional midline spinal exposure, minimally invasive methods of access have been developed. This has led to a flood of minimally invasive retractor and lighting systems as well as percutaneous spinal implants. Although these techniques can be useful as an adjunct, the size of the access required for en-bloc tumour resection means that traditional open approaches are more often utilised.

### 9.3. Image Guidance

Due to the narrow window for placing spinal pedicle screws as well as the marginal excisions required, there have been a number of technological advances in the last 5 years to aid accuracy. Intraoperative navigation has been widely adopted and a system is now available from most of the large implant companies. Like arthroplasty navigation they utilise intraoperative fluoroscopy or CT image devices coupled to reference arrays and navigation cameras to improve accuracy to <1 mm. Software to merge pre-operative MRI and CT scans for tumour purposes has been proven in the brain but still proves problematic for the complex spinal anatomy [171]. 

More recently these navigation principles have been utilised with spinal specific robotic arms to again improve accuracy. With costs prohibitively high their use is only now becoming more widely available and limited to pedicle screw insertion. Further development may allow bony cuts along a trajectory with millimetre accuracy [172], although it has not yet been used for spinal tumour resection.

### 9.4. Augmented Reality (AR)

Exciting advances in augmented reality (AR) have seen the first AR assisted spinal tumour resection at the Johns-Hopkins University in Baltimore using the Xvision system by Augmedics in June 2020 [173]. United States Food and Drug Administration (FDA) approval is currently being sought for augmented reality pedicle screw insertion but its use more widely in spinal tumour surgery is not far away. Multiple overlays on real time anatomy such as blood vessels, neural structures and tumour tissue have the opportunity to improve appropriate resection margins whilst reducing the morbidity and look like the future for spinal tumour surgery.

## 10. Conclusions

OS tumours pose a challenge for orthopaedic surgeons due to their complex anatomical variation, close proximity to critical structures and high risk of recurrence if adequate margins are not achieved [174]. Pre-operative OS imaging has developed massively over recent decades, with a combination of MRI and PET, tumour extent can be better identified, allowing surgeons more confidence in their planning. Both CANS and PSI have been shown to aid the accurate resections of OS, but are both still developing technologies with limitations of their own. A common issue with both technologies is that they must be prepared prior to the surgery, giving the tumour time to change, and are accurate only with regards to bony, and not soft tissue anatomy. Intraoperative imaging of tumours is a developing field and may help combat the above issues. Fluorescent dyes such as indocyanine green are now in clinical use for OS surgery, whilst Cherenkov imaging appears to be an enticing avenue for the development of this field; hopefully this will aid the identification of tumour margins, which remains one of the biggest challenges in OS surgery. Unfortunately, it is yet unknown whether neoadjuvant chemotherapy is compatible with these techniques; necrosis of the OS cells may reduce uptake of the dyes/tracers although viable cells may still be visible. Whilst very much in the early stages, AR has the ability to further enhance this, not only highlighting the tumour, but also highlighting vital structures, hopefully further decreasing morbidity. Meanwhile, the development of the 3D printing materials, as well as a decrease in cost and increase in availability, has the potential to revolutionise implants in OS patients, improving functional outcomes and decreasing the need for reoperation. 

It must also be remembered that these advances must not necessarily be used in isolation. Whilst it may already be clear that techniques such as CANS for resection and the use of 3D printed implants may already go hand in hand, multiple different techniques may be used at once to ensure accurate resection. Take Figure 6 for example, the cut to the superior pubic ramus was made using CANS, whilst custom 3D printed jigs guided the remaining cuts and drilling. Meanwhile, a 3D printed model of the tumour was available in the theatre, allowing the surgeons to cross-reference throughout, and be confident that sufficient resection had occurred. In the future, navigation systems also have the possibility to be combined with robotic systems, aiming to reduce human error further.

In summary, there are a number of exciting developments on the horizon for the surgical management OS. However, as is often the case with rare conditions such as OS, there is a lack of large scale, randomised trials to ascertain which of these technologies produce the best outcomes.

## Figures and Tables

**Table 1:** The most common subtypes of OS [9,10].

Anatomical Location	Subtype		Prevalence
**Intramedullary**	Conventional	Osteoblastic	~40%
		Chondroblastic	~20%
		Fibroblastic	~20%
	Telangiectatic		<4%
	Small cell		1.5%
	Low-grade central		1–2%
**Cortex/surface**	Parosteal		4%
	Periosteal		<2%
	High-grade surface		<1%

**Table 2:** The main materials used for 3D printed modelling and implants. AM = Additive Manufacturing, SLA = Stereolithography, DLP = Digital Light Processing, FDM = Fusion Deposition Modelling, SLS = Selective Laser Sintering, DMLS = Direct Metal Laser Sintering, SLM/DMLM = Selective Laser Melting/Direct Metal Laser Melting, EBM = Electron Beam Melting, PLA = Polylactic Acid, PCL = Polycaprolactone, ABS = Acrylonitrile Butadiene Styrene, PEEK = Polyether Ether Ketone [78,80,81,82,83,84,85,86,87,88].

Material	3D Printing Process	Applications	Pros	Cons
**Photopolymer resins** [78,81,82,83,84]	SLA, DLP	SLA, DLP	- High accuracy - High speed - Low cost - High complexity models - Flexible printing setup	- Low strength and durability - UV sensitive - Limited use in heavy applications
**Polymers:** **PLA, PCL, ABS** [85,86]	FDM	Biodegradable scaffolds Rapid implant prototyping	- Low cost - High speed - Widely accessible - Good structural properties for modelling	- Low accuracy depending on nozzle thickness - Limited to prototyping - Limited strength
**PEEK** [88]		Spinal and cranial 3D printed implants	- High strength - Good abrasion resistance - Elastic modulus close to bone - Highly versatile with other biomaterials - Radiolucent - Stable at high temperatures	- Does not fuse to bone; requires filler or coating to enhance osseointegration - Low rigidity - High cost - Low UV resistance
**Metals:** **Titanium and Ti-alloys (Ti6Al4V); currently the most commonly used material for 3DP implants)** [80]	SLS, DMLS, SLM/DMLM, EBM	Wide variety of 3D printed orthopaedic implants	- High strength and durability - Low weight - Possibility of porous structures allowing for osseointegration - High fatigue resistance - Highly biocompatible	- High cost of manufacturing - Higher elastic modulus than polymers - Low hardness - High notch sensitivity - Relatively poor wear and frictional properties
**Cobalt-chrome and CoCr alloys**			- High strength in load bearing applications - High hardness - Can be highly polished postproduction	- Corrosion resistance lower than Ti-alloys - Wear can produce immunogenic particle - Lower strength: weight ratio that Ti
**Stainless Steel (316L)**			- High strength- High corrosion resistance - Low material cost - High hardness	- High weight - High elastic modulus - Common alloying elements can induce toxicity (Ni, Cr)
**Tantalum** [87]			- High chemical resistance - Highly biocompatible with low toxicity - High corrosion resistance - Modifiable structural surface postproduction	- Limited evidence for clinical use - Very high cost - Lower strength than above metals - Difficult to process using traditional AM techniques

## Data Availability

Not applicable.
